# Roadmap to engagement: Bringing patient partners into cancer research and beyond

**DOI:** 10.1017/cts.2023.602

**Published:** 2023-08-02

**Authors:** Kim S. Kimminau, Cheryl Jernigan, Hope Krebill, Sara Douglas, Jill Peltzer, Jill Hamilton-Reeves, Ronald C. Chen, Roy Jensen

**Affiliations:** 1 Department of Family and Community Medicine, University of Missouri-Columbia, Columbia, MO, USA; 2 University of Kansas Cancer Center, University of Kansas School of Medicine, Kansas City, KS, USA; 3 Masonic Cancer Alliance, Fairway, KS, USA; 4 Patient Advocacy and Engagement Talaris Therapeutics, Louisville, KY, USA; 5 University of Kansas School of Nursing, Kansas City, KS, USA; 6 KU Department of Urology, University of Kansas Medical Center, Kansas City, KS, USA; 7 Department of Radiation Oncology, University of Kansas Cancer Center, Kansas City, KS, USA

**Keywords:** Cancer, patient, engagement model, education, team science

## Abstract

The University of Kansas Cancer Center (KU Cancer Center) initiated an engagement program to leverage the lived experience of individuals and families with cancer. KU Cancer Center faculty, staff, and patient partners built an infrastructure to achieve a patient-designed, patient-led, and research-informed engagement program called Patient and Investigator Voices Organizing Together (PIVOT). This special communication offers an engagement roadmap that can be replicated, scaled, and adopted at other cancer centers and academic health systems. PIVOT demonstrates that collaboration among academic leaders, investigators, and people with a lived experience yields a patient-centered, vibrant environment that enriches the research enterprise.

## Introduction

Patient engagement improves the value of biomedical research and health care delivery [1–7]. In terms of direct impact, integrating patients’ lived experiences has influenced patient-reported outcomes [8], shared decision-making tools [9], self-management care guidance [10], health-quality of life metrics [11], and many other aspects of research.

Even with requirements by funders, routinely designing and executing patient engagement in research has been elusive. A lack of infrastructure to address opportunities to improve patient-, caregiver-, and family-centered engagement drove the University of Kansas Cancer Center to create a solution designed to accelerate engagement and impact research. Although there have been models of engagement for various specific cancers [12–16], we found no published reports of an organized, system-level engagement infrastructure model to advance comprehensive patient engagement in research at academic cancer centers. NCI-designated cancer centers are charged with community engagement to create bidirectional relationships to inform research and services [17]. The funding opportunity, released in 2016 (PAR-17-095) and reissued in 2020 (PAR 20-043), offers a springboard for developing an engagement roadmap at NCI-designated cancer centers. This special communication shares how the KU Cancer Center established a patient-driven program that meets the NCI-designated cancer center intent with a sustained infrastructure to conduct patient-centered, cancer research. We reasoned that modeling this in the context of the KU Cancer Center could offer a model for wider-scale adoption and be leveraged by KU’s clinical and translational science award program as well as in other institutes or centers, too.

### Setting the Stage

The University of Kansas Cancer Center determined it would transform its culture to be more inclusive, with a deliberate focus on patient engagement. As described by Marlett *et al*, patient engagement is highly influenced by institutional ideology, as engagement with patients may be considered a challenge to professional expertise and expected role [18]. The process to develop PIVOT included research faculty, cancer center program staff, and patient research advocates approaching the center director to discuss their interest in developing an engagement program. Group discussions and brainstorming resulted in an initial plan that was presented and approved by the KU Cancer Center leadership team. The KU Cancer Center Director allocated funding for a start-up process that provided protected time for staff (Krebill) and center researcher (Kimminau), funds to remunerate patient partners as consultants (i.e., Jernigan), and funds to hire a half-time project manager (Douglas).

### Establishing PIVOT

This roadmap was informed both by the experiences of the KU Cancer Center and by the emerging guidelines for effective patient engagement in research [19]. To avoid tokenism [20], the startup team included a lead patient advocate (Jernigan, KU) Cancer Center staff (Krebill), a lead researcher (Kimminau), and the project manager (Douglas). This team steered the initiation effort. They began by asking KU Cancer Center directors and physicians to identify potential patients, family members, and caregivers or local cancer advocates who they thought would be open to the idea of receiving training, co-developing training materials, working with investigators, and helping establish an engagement program. This intentionally avoided the startup process being dominated by any one cancer type. They also identified candidates from community leaders, community organizations (e.g., Gilda’s Club, local Komen Affiliate, etc.), community hospitals, and standing University of Kansas Medical Center community advisory boards (e.g. *JUNTOS* [21], Faith Works [22]). While not meant to mirror the KU Cancer Center’s patient demographic profile, the team invited individuals with attention to diversity accounting for race, ethnicity, socioeconomic status, geography (especially with respect to including rural participants), education attainment, and cancer type and cancer experience. Those selected formed the Patient Partner Development Team. The intent was to have this group establish the framework for PIVOT.

**Figure 1.**
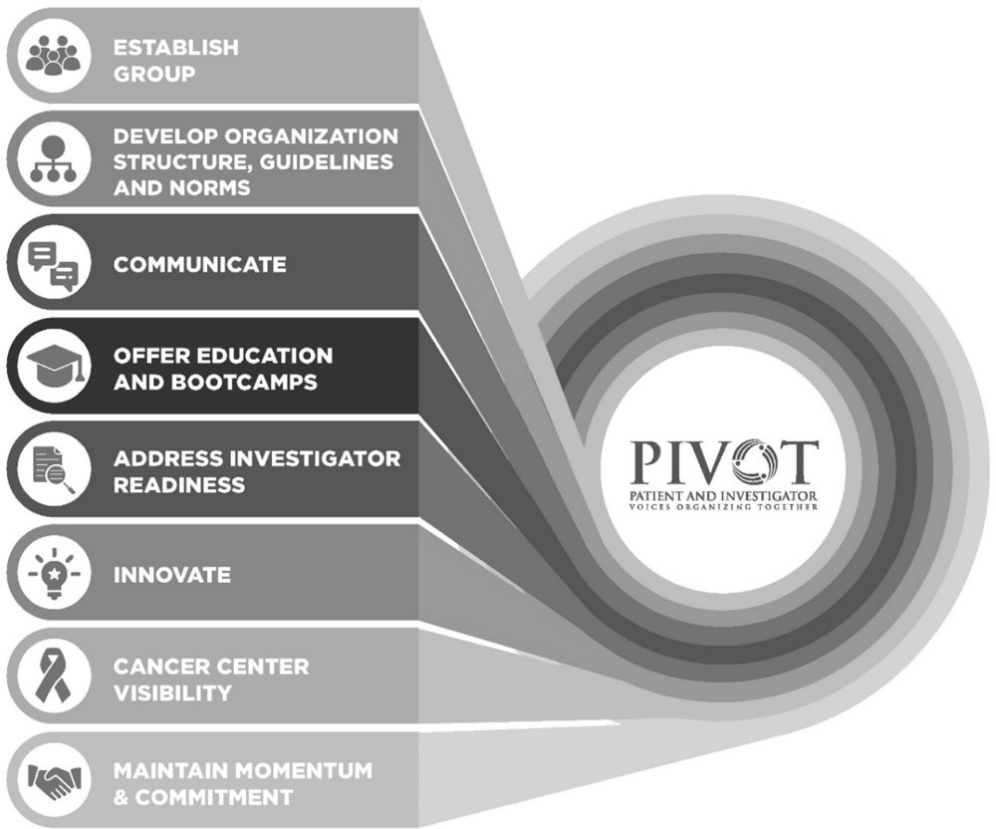
Patient and investigator voices organizing together (PIVOT) roadmap.

Fig. 1 provides an overview of PIVOT’s roadmap elements. The Patient Partner Development Team created the initial infrastructure for PIVOT. Once PIVOT was launched, PIVOT formed a revised Patient Leadership Team (PLT) to serve as a smaller decision-making group, led by the lead patient research advocate (Jernigan). The PLT determined PIVOT’s new member recruitment strategy; communication approaches including web, social media, and internal communication preferences; and education priorities/curriculum needed to level-set members’ knowledge about research generally and cancer research, specifically.

The lead patient research advocate chairs group meetings of the PLT. It is important for members to consistently see a patient in the primary leadership role. This demonstrates and reinforces the authenticity of commitment to patient voice and priorities driving PIVOT’s agenda and operation. Meetings happen during early evening hours to accommodate work schedules and other obligations. Members are paid hourly based on PCORI and other funder remuneration guidelines ($60/hour). Center staff and the lead researcher attend PLT meetings to assist in facilitation and manage minutes and tasks. Their presence also assisted in consistent communications with center leaders to offer updates, share needs, and discuss PLT suggested opportunities for collaboration.

The PLT discussed and then codified meeting norms and values (Table 1). These emerged from facilitated short, targeted strategic planning sessions. PIVOT members were eager to hear each other’’s perspectives, and the facilitator used appreciative inquiry [23,24] to engage the group and achieve consensus.

**Table 1.** Patient and Investigator Voices Organizing Together (PIVOT) operating values and guidelines

1	Come to the effort fully accepting that patients must truly, authentically lead.
2	Plan to ensure diversity in as many areas and at as many inclusive levels as possible.
3	Move quickly to establish a team culture and transparent, operational ground rules.
4	Encourage innovation and problem solving (i.e., encourage participants to develop committee structures, committee charges, chose the duration of work) and adjust tempo appropriate for participants.
5	Take every chance to acknowledge the generosity of participants – both patient and researcher – who are willing to get involved.
6	Budget to include funds for project management, patient leaders, Rapid Reactor Team participation, and other staff. The obligation must be funded to be sustained or the engagement effort will risk losing credibility. Always recognize patient participants in as many ways as is feasible.
7	Accept that engagement is an ongoing process that offers a mutually reinforcing learning environment. Admit failure or shortcomings during the process and be transparent and honest about them with everyone.
8	Keep your focus on patient participants and investigators and be creative with how to leverage their enthusiasm and engagement.
9	Take the path most likely to result in authentic engagement. Make change where change can happen – be pragmatic.
10	Cancer center leadership must “walk the talk” regarding engagement especially with demonstrated, heartfelt, authentic, and visible commitment by the director and other leaders.

PIVOT was established as a part of the community outreach and engagement efforts of the KU Cancer Center. To improve visibility, PIVOT developed a unique, complementary logo. An online service developed logo options within the visual context of the KU Cancer Center’’s brand identity, and the PLT voted to select the final version. Other examples of the Cancer Center’’s support of PIVOT communications include a dedicated PIVOT web page and the prominent display of PIVOT materials at KU Cancer Center-sponsored meetings.

The primary purpose of PIVOT is to promote patient engagement while serving as a resource for all cancer investigators to conduct patient-centered, patient-informed research. Fidelity to this purpose guided PIVOT’’s investment in research education. This crucial step in the roadmap is informed in the spirit of the Boot Camp Translation model [25,26]. Patient partners shared that they needed tailored, plain language education to enable them to participate effectively and gain confidence to collaborate with researchers. The bootcamp’s aim is to strike a balance between covering enough basic and clinical science and research methodology to increase participants’ confidence, but not so much as to be overwhelming. PIVOT members helped to select bootcamp topics. PIVOT members also selected recruitment strategies to engage an ever-widening group of participants (Table 2). While the effort started on a relatively small scale (20 participants), each subsequent bootcamp increases PIVOT by an average of 25 new members. Since inception, more than 100 patients and family members have attended a PIVOT bootcamp (Table 3).

**Table 2.** PIVOT educational resources and training materials

Resource/Training	Topics							
*PIVOT Boot Camp: A Journey into Patient Research (1/2 day session)*	Welcome & Patient Introduction	Importance of Patient Research Advocates at the University of Kansas Cancer Center	Introduction to Cancer Research	Cancer Biology Lab Tours	Understanding Patient Research Advocacy	PIVOT Program Introduction and How to Get Involved	Rapid Reactor Team (RRT) Simulation and Workgroups	Engaging the Broader Community
*Webinars/YouTube Resources*	Member Testimonials	Creating an NIH Advocate Biosketch	How to Tell Your Cancer Story in 5 Minutes or Less	PIVOT Rapid Reactor Team (RRT) Introduction	Informed Consent Review Training	Grant Review Training	Translational Research Training Drug Discovery and Development at KU Cancer Center	What’s Basic Science/research? Why is it Important? How Does it Fit into the Scheme of Things?
*PIVOT Advocate / Researcher Working Together Toolkit*	Building Advocate-Researcher Relationships to Strengthen Research	Guidelines for Advocate Involvement in KU Cancer Center Research	Patient Advocate Involvement Plan – Suggestions for Researchers	Researcher and Advocate Responsibilities by Project Stage	Resources for Advocates: Patient Letter of Support, Patient Biography Template	Writing a Lay Abstract Together		
*Online Training Modules*	Getting Started: The Basics of Patient Research Advocacy	The University of Kansas Cancer Center	Cancer & Cancer Center Basics	What is Research Advocacy?	Role and Engagement of Patient Research Advocates	Issues Important to Patient Research Advocates		

**Table 3.** PIVOT research advocate demographics

Characteristic	Number/Percent
*Race*	
White	59/85 (70%)
African American/Black	17/85 (20%)
American Indian /Alaska Native	3/85 (4%)
Asian/Pacific Islander	3/85 (4%)
Other	3/85 (4%)
*Sex*	
Female	72/83 (87%)
Male	11/83 (13%)
*Ethnicity*	
Hispanic	4/81 (5%)
Non-Hispanic	76/81 (95%)
*Age*	
18-40	21/114 (18%)
41-60	58/114 (51%)
61-90	36/114 (31%)
*Member County RUCA Codes*	
1	111/131 (85%)
2	12/131 (9%)
3	2/131 (1.5%)
4	2/131 (1.5%)
5	1/131 (0.7%)
7	1/131 (0.7%)
10	2/131 (1.5%)

RUCA codes are based on 2010 Rural-Urban Commuting Area Codes - 1, Metropolitan area core: primary flow within an urbanized area (UA); 2, Metropolitan area high commuting: primary flow 30% or more to a UA; 3, Metropolitan area low commuting: primary flow 10%–30% to a UA; 4, Micropolitan area core: primary flow within an Urban Cluster of 10,000 to 49,999 (large UC); 5, Micropolitan high commuting: primary flow 30% or more to a large UC.

We conducted four focus group sessions with 15 KU Cancer Center investigators from across the translational science spectrum to ask them about patient engagement in research. Not surprisingly, participants had varying levels of engagement experience. Most investigators reported they were more likely to engage patients *after* a study had begun. Mostly, they sought input about participant recruitment and retention. Generally, the investigators viewed patient engagement positively. However, they lacked an appreciation, particularly among those in the basic sciences, of the various ways patients could inform their research. Across all four focus groups, there was a lack of readiness for engagement. They shared that they and their colleagues do not have the time for engagement or for the engagement training they assumed was necessary to gain competency. Investigators were opposed to mandatory training because they felt it would be counterproductive and a barrier to facilitating engagement. Interestingly, focus group participants were more comfortable sharing what training should be required for patient research advocates, and how to acknowledge and compensate advocates for their contribution, than to identify their own training needs. PIVOT and KU Cancer Center leaders overestimated the readiness of researchers to become involved in patient-engaged research. Investigators’ assumptions that engagement takes too much time, patients would not be able to be helpful, and worries about slowing research processes dissuaded them from welcoming the opportunities PIVOT sought to offer.

PIVOT leaders decided to incrementally improve bidirectional engagement by developing the “Rapid Reactor Team” (RRT). The RRT is a way for researchers to spend time with bootcamp-trained PIVOT research advocates and reap engagement benefits in a short, one-hour design studio [27]-styled session. PIVOT provides a blank, five PowerPoint slides template that the researcher is required to use to describe their study. PIVOT members interested in the topic volunteer to participate (eight to 10 participants maximum: ideally 5–7). The researcher submits their PowerPoint slides in advance to receive revision suggestions and coaching, usually on thinning their slide deck and on the need to use plain language. Ideally, revised slides are shared with the volunteer PIVOT members planning to attend the session. During the one-hour session, the researcher presents their slides for the first 10–15 minutes. After the presentation, the balance of the hour is reserved for PIVOT member reactions, questions, input, and discussion. The session is moderated by a facilitator to assist an effective bidirectional conversation that sometimes calls for research translation or the facilitator asking the researcher to restate or explain some element of their presentation to address a PIVOT member’s question more clearly. Each PIVOT participant is asked and encouraged to make comments, and they can follow-up with written comments after the session, too, if they prefer, with their input for the researcher.

An unanticipated benefit of the RRT model is that it proved to be an effective way to engage new PIVOT members. These sessions offer a low-stress, yet stimulating, way to learn about cancer research. Based on overwhelmingly positive reception, PIVOT added the RRT process into bootcamp training by providing case studies in a mock RRT format. This training gives bootcamp participants the chance to practice responding to research questions and to become familiar with the format.

To further strengthen RRT’s utility, researchers seeking a letter of support for their proposals from patients are encouraged to conduct an RRT session. This process allows PIVOT members to consider whether they agree to support an application. It also provides a way for the researcher to consider patient-informed modifications to their proposal. If the researcher seeks a patient collaborator to join their research team, the RRT group participants are asked first if they have an interest. Other PIVOT members can be identified to make a match based on lived experience and interest, and these individuals often also provide individual letters of support or commitment to participate. Financial support for the RRT PIVOT participants is provided at the same hourly rate as other PIVOT activities and is paid for with KU Cancer Center funding so it does not cost the researcher anything but their preparation and presentation time.

Process evaluation is emerging from the steady increase in the number of PIVOT participants and their duration of commitment to participate while maintaining the foundational commitment to diversity and some degree of balance across cancer types, cancer experience, and catchment area representativeness (Table 3).

**Figure 2.**
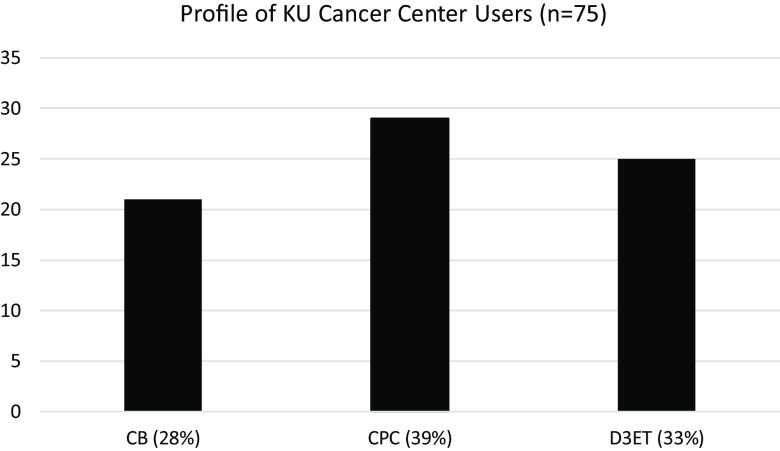
Use of PIVOT by KU Cancer Center investigators. CB = cancer biology; CPC = cancer prevention and control; D3ET=drug discovery, delivery, and experimental therapeutics.

Figure 2 displays RRT use by cancer center program demonstrating not only how positively the RRT has been received but also demonstrates the reach and value of patient engagement in various types of basic, clinical, and translational cancer research.

More difficult to evaluate and measure is culture change stimulated by the establishment of PIVOT. Gaining momentum and shaping an engagement culture in a cancer center hinges on (1) moving along a continuum of increasing exposure and commitment and (2) working toward expanding the evidence that engagement impacts research outcomes [1,28]. PIVOT exposure to the center’s research community began with PIVOT members simply attending the center’s annual scientific meeting without any specific accommodations. Over time, PIVOT members played increasingly visible and important roles at the conference (Table 4). This growth required intentional inclusion of engagement by leadership in both the planning and execution of the center’s events.

**Table 4.** Evolution of PIVOT engagement at annual KUCC meetings. (Table displays year on year additive activities.)

2017	2018	2019	2020
PLT invited to symposium	PLT and PIVOT Task Force members invited to symposium	PIVOT members invited and recognized at KUCC program meetings	Virtual Week-Long Research Symposium included PIVOT members
KUCC Director opened symposium describing PIVOT & recognizing PLT members	Researchers required to submit a 3-line lay summary with abstract	PIVOT meet & greet with attendees during lunch	Symposium planning committee included PIVOT Program Manager
PIVOT submitted abstract & research poster	PLT led a breakout session on PIVOT resources		Pilot grants had a 30-line lay abstract & notation if an advocate was involved
PIVOT hosted an advocate poster walk			Inaugural PIVOT Day
PIVOT researchers recognized with a PIVOT badge ribbon			PIVOT selected six abstracts for mini-Rapid Reactor Team (RRT) sessions
			RRT selected three researchers to receive patient engagement travel awards

Ongoing outcomes evaluation is planned to track the success of research applications that used PIVOT resources, recommendations, and experts; the successful resubmission of applications; and the ongoing connectivity between individual PIVOT research advocates and academic research teams. Impact evaluation to focus on long-term, sustained changes in the KU Cancer Center, will continue to monitor uptake from the novelty of PIVOT to the routine expectation that PIVOT input and collaboration are essential to the center’s success.

### Summary

Patient engagement must be tailored and responsive to the needs of patients [29–32]. As expectations for patient engagement increase, risks of tokenism and even greater marginalization of underrepresented patients increase. Furthermore, engagement is influenced by institutional ideologies, professional attitudes, and patient readiness [18]. Reducing risks of failure requires intentionality and planning [33]. Overcoming risk factors requires sensitivity to constructing a roadmap that accounts for multiple, simultaneous constraints and opportunities. Engagement and genuine support from senior leadership are essential for both growth and sustainability of a program. This roadmap was not determined *a priori*, but instead, it evolved over time through learning what was effective, what patient partners needed, and how to best meet the needs of the cancer research community.

Five resources and skills are essential to pursue the milestones of this roadmap. This list is transferrable to other engagement startup efforts important to other cancer centers, Clinical and Translational Science awardees, and other academic health centers that intend to establish a durable, patient-led, and informed platform designed to be a resource for collaboration.

1. Deploying staff who have program implementation expertise, operational oversight experience, and understanding of the mechanisms to initiate a startup program was crucial. Staff must be able to relate to patients in ways that ensure they feel valuable and recognized for their contributions every time they interact with the program.
2. Having a seasoned patient research advocate with experience in leading initiatives and modeling behaviors for newer patient research advocates was critical. An added benefit was this patient leadership model helped attract additional PIVOT members with experience in engagement. This substantially expanded PIVOT’s leadership capacity.
3. Coaching skills are essential. Coaching and appreciation for adult learning styles offered ways to help patients adjust their behavior to interact effectively in a new role with clinicians and clinical researchers. Coaching offers a dual advantage of (1) assisting in communication and interaction skills for the research advocate, and (2) helping investigators feel less intimidated working directly with patients. For some investigators, exposure to PIVOT members was their first experience talking with someone with a lived experience in the cancer they study.
4. Conducting an environmental scan to find researchers engaged with patient and community partners did not yield substantially new information for PIVOT. However, this step is recommended to ensure current structures are accounted for when developing the model. Also, it is wise to be aware of academic boundaries and biases that may exist among engagement faculty and target communities in advance of initiating the program [34].
5. The entire engagement enterprise requires high touch, humanistic, and skilled facilitation with particular attention to having someone who can distill technical information effectively for non-researchers. Having someone who excels in science communications is invaluable.

Launching an engagement program requires committed leadership. While no memorandum of understanding was established, it was the center’s director who found the case for PIVOT compelling and supported it with resources. He also used his influence to elevate awareness and persuade investigators to utilize PIVOT to bring their own creative ideas and challenging research problems to the group. The commitment to dedicated staffing, sustainable funding, and intentional training programing strengthened the capabilities of patient partner(s) and collaborating researchers to engage. The result so far is a vibrant and impactful program that enhances the quality and patient relevance of the cancer center’s research agenda and outcomes.
